# Healthy adiposity and extended lifespan in obese mice fed a diet supplemented with a polyphenol-rich plant extract

**DOI:** 10.1038/s41598-019-45600-6

**Published:** 2019-06-24

**Authors:** Virginie Aires, Jérôme Labbé, Valérie Deckert, Jean-Paul Pais de Barros, Romain Boidot, Marc Haumont, Guillaume Maquart, Naig Le Guern, David Masson, Emmanuelle Prost-Camus, Michel Prost, Laurent Lagrost

**Affiliations:** 1 University of Bourgogne-Franche-Comté, F-21000 Dijon, France; 2 INSERM U1231 “Lipids, Nutrition, Cancer”, F-21000 Dijon, France; 3 LipSTIC LabEx, F-21000 Dijon, France; 4 Lipidomic Platform, F-21000 Dijon, France; 5 Platform of Transfer in Cancer Biology, Centre Georges-François Leclerc, F-21000 Dijon, France; 6 Department of Biology and Pathology of Tumours, Centre Georges-François Leclerc, F-21000 Dijon, France; 7 LARA-Spiral Laboratories, F-21560 Couternon, France; 8 University Hospital of Dijon (CHU), F-21000 Dijon, France; 9 VITAGORA Competitiveness Cluster, F-21000 Dijon, France

**Keywords:** Lipids, Ageing, Fat metabolism, Metabolic syndrome

## Abstract

Obesity may not be consistently associated with metabolic disorders and mortality later in life, prompting exploration of the challenging concept of healthy obesity. Here, the consumption of a high-fat/high-sucrose (HF/HS) diet produces hyperglycaemia and hypercholesterolaemia, increases oxidative stress, increases endotoxaemia, expands adipose tissue (with enlarged adipocytes, enhanced macrophage infiltration and the accumulation of cholesterol and oxysterols), and reduces the median lifespan of obese mice. Despite the persistence of obesity, supplementation with a polyphenol-rich plant extract (PRPE) improves plasma lipid levels and endotoxaemia, prevents macrophage recruitment to adipose tissues, reduces adipose accumulation of cholesterol and cholesterol oxides, and extends the median lifespan. PRPE drives the normalization of the HF/HS-mediated functional enrichment of genes associated with immunity and inflammation (in particular the response to lipopolysaccharides). The long-term limitation of immune cell infiltration in adipose tissue by PRPE increases the lifespan through a mechanism independent of body weight and fat storage and constitutes the hallmark of a healthy adiposity trait.

## Introduction

Obesity is characterized by the accumulation of adipose tissue and has long been associated with a significantly higher all-cause mortality rate, mostly due to metabolic diseases and cardiovascular complications^1^. However, obesity is not consistently associated with metabolic disorders, prompting the challenging concept of healthy or benign obesity that might not translate into obesity-associated disorders and mortality later in life^2,3^. Although recent studies have reported an increased risk of cardiovascular diseases in obese individuals with no metabolic abnormalities compared with lean individuals with no metabolic risk factors^4^, the risk is still lower than in individuals with unhealthy obesity. In fact, the healthy obesity concept remains an open question in light of (1) the harmful consequences of adipose tissue hypertrophy that may vary between individuals, (2) increased numbers of metabolic abnormalities that still occur in some lean individuals, (3) the lack of typical metabolic obesity-associated complications in some overweight subjects, and (4) the identification of tissue-specific gene co-expression networks that allow researchers to distinguish between healthy and unhealthy obese individuals^5–7^. In addition to observational studies in humans, a paucity of experimental data unambiguously support a direct and causal relationship between adipose tissue dysfunction, metabolic disorders and life expectancy. In other words, researchers have not established whether healthy obesity exists and translates into fewer metabolic abnormalities, better health and increased lifespan independent of adiposity compared to unhealthy obesity, and the cellular and molecular mechanisms must be deciphered. These questions require specifically designed experimental models in which the adipose tissue accumulation and composition are controlled and monitored. Although recent overfeeding studies in zebrafish provided some evidence in favour of the development of metabolically heterogeneous obesity independent of adipocyte hypertrophy, the molecular and cellular mechanisms that would predispose an individual to pathological and unhealthy obesity have not been completely elucidated^8^. To our knowledge, only a resveratrol treatment has been reported to increase the survival of obese mice without reducing body weight^9^.

The composition of the adipose tissue is one of the putative underlying mechanisms that might determine the pathogenic nature of obesity, which is the focus of the present study. Importantly, adipose tissue is heterogeneous in nature, both from structural and metabolic perspectives^10–13^. In addition to adipocytes, immune cells colonize the adipose tissue and subsequently trigger inflammation, which is currently recognized as a leading component of pathogenic obesity. Therefore, in addition to adipose tissue hypertrophy and lipid accumulation, infiltrating immune cells might actually constitute the harmful components of the low-grade inflammation and its consequences in obesity. Elevated endotoxaemia, resulting in particular from dysbiosis and gut barrier dysfunction, has been shown to contribute significantly to the accumulation of immune cells in adipose tissue, as well as low-grade inflammation and its harmful consequences^14,15^.

A primary goal of the present study was to assess the impact of obesity induced by the consumption of an experimental diet on the adipose tissue composition and longevity in mice fed a high-fat/high-sucrose (HF/HS) diet compared to healthy, control mice fed standard show (Std). A second goal was to determine whether metabolic dysfunction and abnormalities in the adipose tissue composition are preventable, despite the persistence of adipose tissue hyperplasia and to elucidate the consequences on health and longevity. Importantly, in the present study, the healthy obesity trait was strictly characterized by a lack of change in adiposity, fat storage in the adipose tissue and adipocyte size. In addition, the present study not only examined risk factors and comorbidities (which may eventually develop into diseases) but also the obesity-related decrease in lifespan that has been considered here as the ultimate trademark of an unhealthy phenotype throughout life^16^. Because even a minimal weight loss was previously shown to be sufficient to reduce obesity-related disorders, a major requirement here was to maintain identical elevated weights in control and treated obese groups throughout the study to explore adipose tissue function (with a specific emphasis on immune cell infiltration and lipid metabolism) in the absence of confounding factors. This strategy allowed us to revisit the myth of innocent obesity^16^ in a well-controlled model of diet-induced obesity.

Herein we show that independent of weight loss, the long-term limitation of immune cell infiltration in mouse adipose tissue by a polyphenol-rich plant extract effectively prevents adipose tissue dysfunction and reduction in the median lifespan.

## Results

### Obese mice display a shorter median lifespan

**Figure 1:**
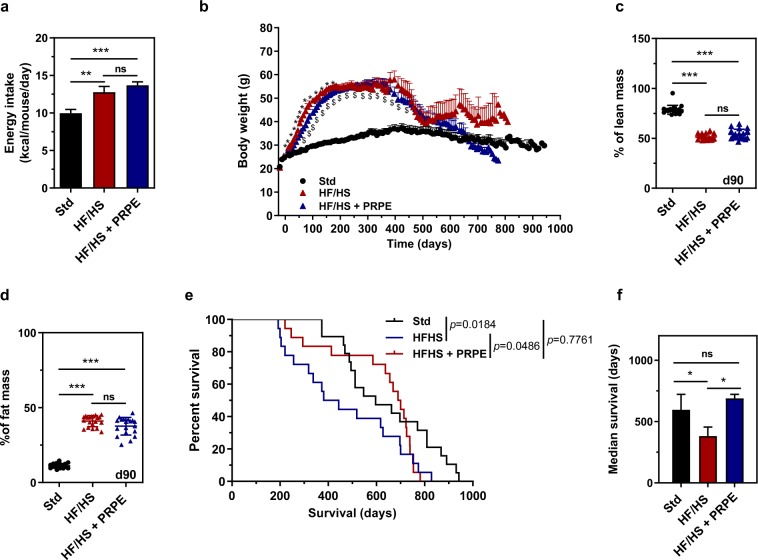
HF/HS diet supplementation with PRPE increases the median lifespan independent of body fat mass. (**a**) Food intake was measured throughout the lifespan of mice fed a standard show (Std), a high-fat/high sucrose diet (HF/HS) or a HF/HS diet supplemented with a polyphenol-rich plant extract (PRPE; HF/HS + PRPE), and daily energy intake (Kcal/mouse/day) was calculated. Data are presented as the means ± s.e.m. and were analysed using the Kruskal-Wallis test with Dunn’s post hoc analysis, n = 13 mice per diet group. **p < 0.01 and ***p < 0.001. (**b**) Changes in the body weight of mice fed the Std diet, a HF/HS diet or a HF/HS diet + PRPE *ad libitum*. Data are presented as the means ± s.e.m. and were analysed using two-way ANOVA followed by Tukey’s post hoc analysis, n = 19 for the Std group and n = 18 for the HF/HS and HF/HS + PRPE groups. *p < 0.001 for the comparison of HF/HS-fed and HF/HS + PRPE-fed mice. ^$^*p* < 0.001 for the comparison of mice fed the HF/HS diet or HF/HS diet + PRPE compared with Std-fed mice. (**c**,**d**) The lean and fat masses of mice were assessed by using EchoMRI^TM^ after 90 days of supplementation. Data are presented as the means ± s.e.m. and were analysed using the Kruskal-Wallis test with Dunn’s post hoc analysis; n = 20 mice each in the Std and HF/HS groups, and n = 19 mice in the HF/HS + PRPE group. ***p < 0.001. (**e**) Kaplan-Meier cumulative survival plots for mice fed either the standard chow (n = 19), HF/HS (n = 18) or HF/HS + PRPE (n = 18) diet. **f)** The median survival (days) ± s.e.m. of mice in each group is shown, and the *p*-value was assessed by using the Peto-Peto Prentice test. *p < 0.05.

Wild-type C57BL/6Rj male mice aged 8 to 12 weeks were randomly assigned to a standard chow or a high-fat/high-sucrose diet *ad libitum* (Table 1). As shown in Fig. 1, a significantly higher total daily mean energy intake was measured throughout the lifespans of HF/HS-fed mice than in Std-fed mice (Fig. 1a). As early as 2 weeks after the initiation of the dietary intervention, HF/HS feeding was associated with a more pronounced and faster increase in body weight than standard feeding (Fig. 1b). The mean maximal weight, which was achieved after 200 to 400 days of administration of the dietary intervention, was 61.32 ± 1.19 and 38.34 ± 1.54 grams in HF/HS-fed obese mice and Std-fed lean mice, respectively (*p* < 0.001, Mann-Whitney U-test). In further support of the obese trait associated with the HF/HS diet, obese mice showed a significant 4- to 5-fold increase in the fat mass percentage and a 2-fold decrease in the lean mass percentage compared to lean mice (Fig. 1c,d). An analysis of survival curves revealed that HF/HS feeding markedly and significantly shortened the median lifespan, with values of 596 and 381 days for lean (Std) and obese (HF/HS) mice, respectively (*p* = 0.0184, Peto-Peto Prentice test; Fig. 1e,f).

**Table 1:** Diet composition.

Ingredients	Chow (Std)		HF/HS		HF/HS + PRPE	
	g/kg	Kcal	g/kg	Kcal	g/kg	Kcal
Casein, 80 Mesh	200	800	200	800	200	800
L-cysteine	3	12	3	12	3	12
Corn starch	575	2300	0	0	0	0
Maltodextrin 10	125	500	93.8	375.2	93.8	375.2
Sucrose	0	0	100	400	100	400
Cellulose, BW200	50	0	50	0	50	0
Soybean oil	25	225	25	225	25	225
Lard	20	180	245	2205	245	2205
Mineral mix, S10026	10	0	10	0	10	0
Dicalcium phosphate	13	0	13	0	13	0
Calcium carbonate	5.5	0	5.5	0	5.5	0
Potassium citrate, 1 H2O	16.5	0	16.5	0	16.5	0
Vitamin mix, V10001	10	40	10	40	10	40
Choline bitartrate	2	0	2	0	2	0
PRPE	0	0	0	0	3.72	0
FD&C yellow dye #5	0.025	0	0.025	0	0.05	0
FD&C red dye #40	0.025	0	0.025	0	0	0
FD&C blue dye #1	0	0	0.025	0	0	0

### Effect of HF/HS feeding on plasma biological parameters

**Figure 2:**
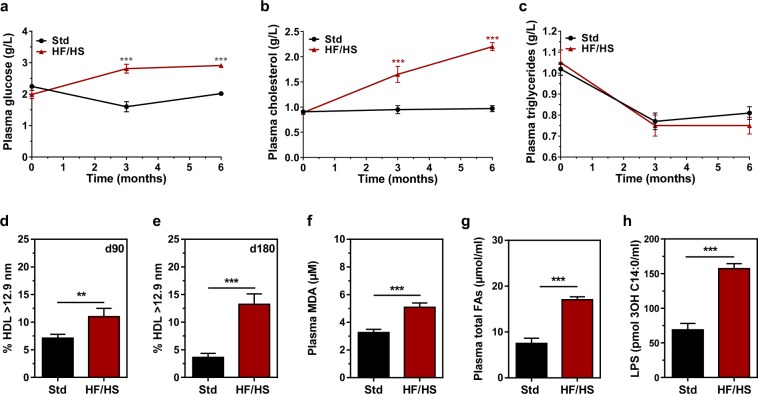
Blood parameters are altered by the HF/HS diet. Plasma glucose (**a**), cholesterol (**b**) and triglyceride levels (**c**) were measured in mice fed either the chow diet (Std) or the high-fat/high sucrose diet (HF/HS), after 0, 3 and 6 months of supplementation. Data are presented as the means ± s.e.m. and were analysed using ANOVA; n = 20 (Std) and n = 20 (HF/HS) per time point. ***p < 0.001. The size distribution of the plasma HDL lipoproteins was assessed by using electrophoresis on Spiragel™ 1.5–25% in mice fed either the chow diet (Std) or the high-fat/high sucrose diet (HF/HS) after 90 (**d**) and 180 (**e**) days of supplementation. Data are presented as the means ± s.e.m. and were analysed using Student’s unpaired t-test; n = 20 (Std) and n = 19 (HF/HS) per time point. **p < 0.01 and ***p < 0.001. (**f**) Plasma MDA levels in mice fed either the chow diet (Std) or the high-fat/high sucrose diet (HF/HS) were measured after 180 days of supplementation. Data are presented as the means ± s.e.m. and were analysed Student’s unpaired t-test; n = 20 (Std) and n = 14 (HF/HS). ***p < 0.001. Plasma total fatty acid (**g**) and lipopolysaccharide (**h**) levels in mice fed either the chow diet (Std) or the high-fat/high sucrose diet (HF/HS) were assessed by LC-MS/MS after 180 days of supplementation. Data are presented as the means ± s.e.m. and were analysed by using the Mann-Whitney U-test; n = 20 (Std) and n = 20 (HF/HS). ***p < 0.001.

As shown in Fig. 2, HF/HS-fed mice displayed significantly higher plasma glucose (Fig. 2a) and cholesterol levels (Fig. 2b) than Std-fed mice, with no difference in plasma triglyceride concentrations (Fig. 2c). Most strikingly, significant amounts of cholesterol accumulated in large-sized high-density lipoproteins (HDL), which contained 3- to 4-fold more cholesterol in HF/HS-fed mice than in Std-fed mice 180 days after the initiation of the dietary intervention (Fig. 2d,e). In addition to hyperglycaemia and hypercholesterolaemia, HF/HS feeding also dramatically increased oxidative stress (as shown by approximately 2-fold higher plasma malondialdehyde (MDA) levels; Fig. 2f), lipid intolerance (as shown by 2-fold higher plasma total fatty acids (FA) levels; Fig. 2g) and endotoxaemia (as shown by 2-fold higher plasma 3-hydroxymyristate (3-OH C14:0) levels; Fig. 2h) compared to Std-fed mice.

### Characterization of the adipose tissue of obese mice

**Figure 3:**
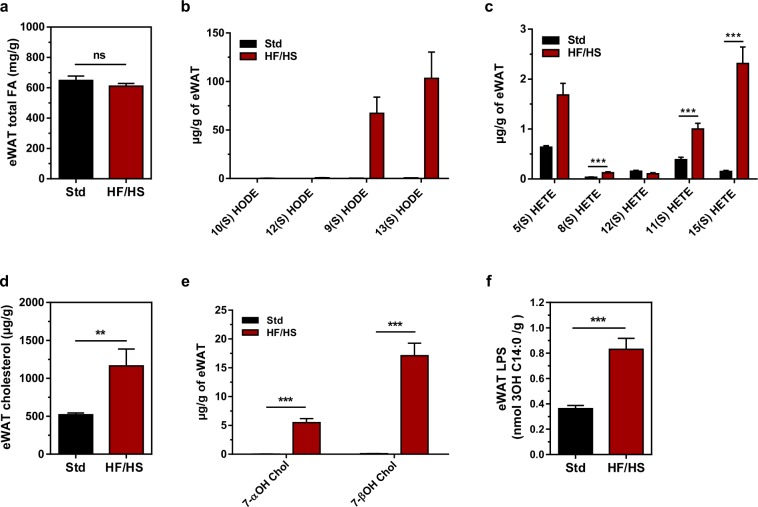
Mice fed the HF/HS diet exhibit an accumulation of cholesterol and cholesterol/lipid oxides in eWAT. (**a**) eWAT total fatty acids in mice fed either the chow diet (Std) or the high-fat/high sucrose diet (HF/HS) for 180 days. Mann-Whitney U-test, n = 20 (Std) and n = 20 (HF/HS). HODEs (**b**) and HETEs (**c**) were measured by LC-MS/MS in eWAT from mice fed either the chow diet (Std) or the high-fat/high sucrose diet (HF/HS) diet for 180 days. Data are presented as the means ± s.e.m. and were analysed using multiple Student’s unpaired *t*-tests; n = 20 (Std) and n = 20 (HF/HS). ***p < 0.001. The levels of cholesterol (**d**) and 7-hydroxycholesterols (7-α-hydroxycholesterol, 7α-OH Chol and 7β-hydroxycholestrol, 7-β-OH Chol) (**e**) in the eWAT of mice fed either the Std or HF/HS diet were measured using GC/MS. Data are presented as the means ± s.e.m. and were analysed using multiple Student’s unpaired *t*-tests; n = 20 (Std) and n = 20 (HF/HS). **p < 0.01 and ***p < 0.001. (**f**) eWAT Lipopolysaccharide (LPS) levels in the eWAT of mice fed either the Std or HF/HS diet. Data are presented as the means ± s.e.m. and were analysed using multiple Student’s unpaired *t*-tests, n = 20 (Std) and n = 20 (HF/HS). ***p < 0.001.

**Figure 4:**
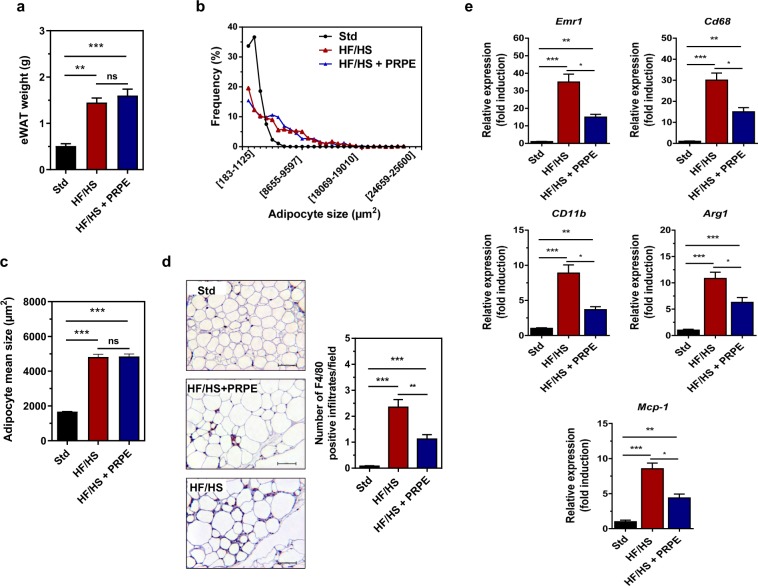
Supplementation of the diet with PRPE reduces HF/HS-induced eWAT cholesterol accumulation and macrophage infiltration. (**a**) eWAT weight of mice fed either the Std, HF/HS or HF/HS + PRPE diet. Data are presented as the means ± s.e.m. and were analysed using the Kruskal-Wallis test with Dunn’s post hoc analysis; n = 20 animals in each diet group. **p < 0.01 and ***p < 0.001). (**b**) Frequency distribution of adipocyte sizes in eWAT from mice in the Std, HF/HS, and HF/HS + PRPE groups. (**c**) Mean adipocyte sizes (µm^2^) in mice from groups fed the Std, HF/HS or HF/HS + PRPE diet. Data are presented as the means ± s.e.m. and were analysed using the Kruskal-Wallis test with Dunn’s post hoc analysis; n = 10 mice per diet group. ***p < 0.001. (**d**) Immunohistochemical (IHC) detection of the macrophage-specific antigen F4/80 in eWAT from Std-, HF/HS- and HF/HS + PRPE-fed mice (20 × magnification, scale bar = 50 µm). Representative images are shown. IHC for F4/80 was quantified by counting F4/80-positive infiltrates in 5 different fields per sample from 2 different sections per sample. Data are presented as the means ± s.e.m. and were analysed using the Kruskal-Wallis test with Dunn’s post hoc analysis; n = 10 animals per diet group. **p < 0.01 and ***p < 0.001. (**e**) Relative mRNA expression levels of murine macrophage markers in mouse groups after 180 days of the diet intervention. *36B4* was used as a housekeeping gene to calculate the ΔCt. Data are presented as the mean fold changes ± s.e.m. calculated with the 2^−ΔΔCt^ method. Data were analysed using the Kruskal-Wallis test with Dunn’s post hoc analysis; n = 20 animals per diet group. *p < 0.05, **p < 0.01, and ***p < 0.001.

Lipid analyses revealed substantial differences in the adipose tissue composition of mice in the HF/HS and Std groups. Although the total fatty acid content per adipose tissue weight did not differ between mice fed the HF/HS and Std diets (Fig. 3a), the adipose tissue from HF/HS-fed mice contained more oxidized fatty acids (Fig. 3b,c), cholesterol, cholesterol oxides (Fig. 3d,e), and endotoxins (Fig. 3f) than the adipose tissue from Std-fed mice. A morphological analysis of hypertrophic adipose tissue in obese mice fed the HF/HS diet revealed enlarged adipocytes (Fig. 4a,d) surrounded by a greater number of infiltrating macrophages (Fig. 4d), accounting for a higher expression of macrophage markers (Fig. 4e) than in lean mice fed the Std diet.

### Polyphenol-rich plant extract extends the median lifespan of obese mice

Wild-type C57BL/6Rj male mice aged 8 to 12 weeks were fed the HF/HS diet supplemented with or without a polyphenol-rich plant extract (PRPE) containing 25% of polyphenols *ad libitum* (Table 1). As shown in Fig. 1a, supplementation of the HF/HS diet with 0.48% PRPE did not modify food intake, with similar daily energy intake values measured in the HF/HS and HF/HS + PRPE subgroups throughout the animals’ lifespans. Supplementation of the HF/HS diet with PRPE was associated with a slight but significantly slower body weight gain in the early phase than in mice fed the non-supplemented HF/HS diet (Fig. 1b). However, the mean maximal weight, which was achieved after 200 days, was identical in the HF/HS and HF/HS + PRPE groups (61.32 ± 1.19 and 60.02 ± 1.08 grams in obese mice fed the HF/HS diet and obese mice fed the HF/HS diet + PRPE, respectively; ns, Mann-Whitney U-test); similar results were obtained for the lean and fat mass percentages (Fig. 1c,d). After 600 days, elderly mice fed a diet supplemented with PRPE lost significantly more weight than elderly mice fed the HF/HS diet alone (Fig. 1b). Strikingly and despite the presence of similar obese traits at day 300, an analysis of survival curves revealed that PRPE supplementation markedly and significantly increased the lifespan, with median lifespan values of 689 and 381 days for obese mice fed the HF/HS diet supplemented with or without PRPE, respectively (*p* = 0.0418, Peto-Peto Prentice test; Fig. 1e,f).

### PRPE improves plasma biological parameters

**Figure 5:**
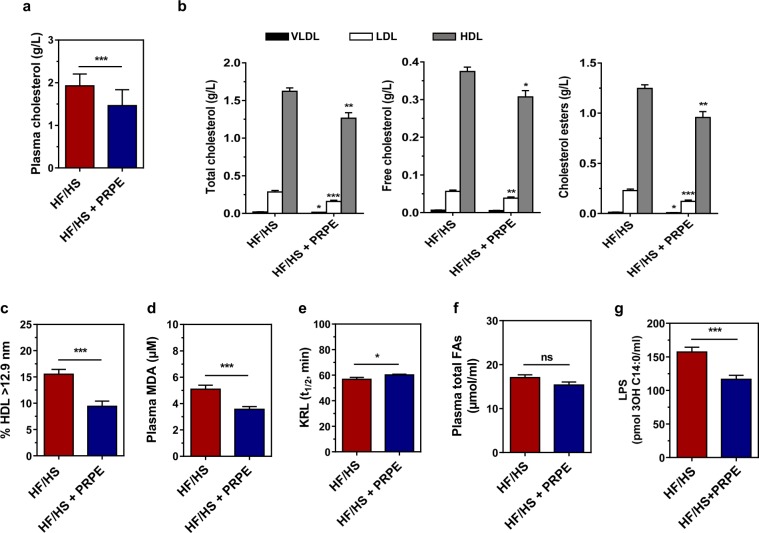
Supplementation of the HF/HS diet with PRPE normalizes blood parameters. (**a**) Total cholesterol levels in plasma from mice fed the HS/HS or HF/HS + PRPE diet *ad libitum* for 180 days were measured using GC/MS. Data are presented as the means ± s.e.m. and were analysed using the Mann-Whitney U-test; n = 19 (HF/HS) and n = 19 (HF/HS + PRPE). (**b**) FPLC-separated lipoprotein fractions from the sera of fasted HS/HS- or HF/HS + PRPE-fed mice were quantified by using electrospray ionization tandem mass spectrometry (ESI-MS/MS). Data are presented as the means ± s.e.m. and were analysed using multiple Student’s unpaired *t*-tests, n = 20 (HF/HS) and n = 20 (HF/HS + PRPE). *p < 0.05, **p < 0.01, and ***p < 0.001. (**c**) Size distributions of plasma HDL in mice fed either the HS/HS or HF/HS + PRPE diet for 180 days were assessed by using electrophoresis on a 1.5–25% Spiragel™. Data are presented as the means ± s.e.m. and were analysed by using Student’s unpaired t-test, n = 20 (HF/HS) and n = 20 (HF/HS + PRPE). ***p < 0.001. (**d**) MDA plasma levels in mice from each group were measured after 180 days of the dietary intervention. Data are presented as the means ± s.e.m. and were analysed using Student’s unpaired t-test; n = 20 (HF/HS) and n = 20 (HF/HS + PRPE). ***p < 0.001. (**e**) Total antioxidant activity was assessed in blood from mice fed either the HF/HS or the HF/HS + PRPE diet for 180 days. Data are presented as the means ± s.e.m. and were analysed by using Student’s t-test, n = 14 (HF/HS) and n = 18 (HF/HS + PRPE). Plasma total fatty acid (**f**) and lipopolysaccharide (**g**) levels in mice from each group were assessed by using LC-MS/MS after 180 days of supplementation. Data are presented as the means ± s.e.m. and were analysed using the Mann-Whitney U-test; n = 20 (HF/HS) and n = 20 (HF/HS + PRPE). *p < 0.05 and ***p < 0.001.

Supplementation of the HF/HS diet with PRPE resulted in significantly lower plasma cholesterol levels (Fig. 5a). The total, unesterified and esterified cholesterol contents of individual plasma lipoprotein fractions (*i.e*., very low-density lipoproteins (VLDL), low-density lipoproteins (LDL) and HDL fractions isolated by using gel permeation chromatography) were significantly lower in mice fed the diet supplemented with PRPE than in mice fed the non-supplemented diet, and the change was particularly prominent in the HDL fraction (Fig. 5b). Coincidently, the plasma HDL subfraction with the largest size (mean diameter greater than 12.9 nm, as determined using native gradient gel electrophoresis) was significantly less abundant after PRPE supplementation (Fig. 5c). Importantly, compared to HF/HS feeding, supplementation with PRPE produced a significant reduction in the levels of the oxidative stress marker MDA (Fig. 5d), a significant increase in the blood antioxidant defences (as evidenced by an extended KRL^TM^ half-life; Fig. 5e), and a significant decrease in endotoxaemia (Fig. 5g). Supplementation of the HF/HS diet with PRPE had no effect on plasma total fatty acid levels (Fig. 5f).

### The beneficial effects of PRPE do not affect the fatty acid composition

**Figure 6:**
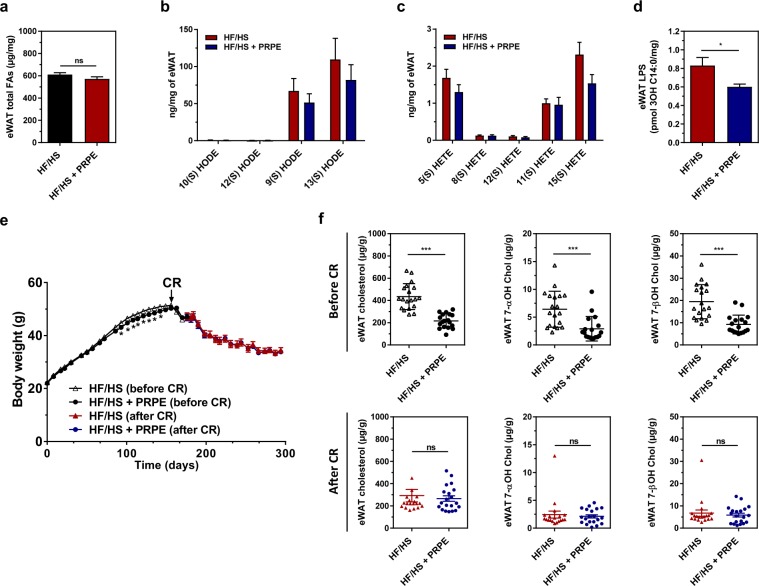
Mobilization of lipids in adipose tissues by caloric restriction. The levels of total fatty acids **(a)**, HODEs (**b**) and HETEs (**c**) were measured in the eWAT from mice fed mice fed either the HF/HS or HF/HS + PRPE diet for 180 days by using LC-MS/MS. Data are presented as the means ± s.e.m. and were analysed using the Mann-Whitney U-test, n = 20 (HF/HS) and n = 20 (HF/HS + PRPE) for **a)** and multiple Student’s unpaired *t*-tests; n = 20 (Std) and n = 20 (HF/HS) for (**b**,**c**). (**d**) Lipopolysaccharide (LPS) levels in the eWAT of mice fed either the HF/HS or HF/HS + PRPE diet for 180 days. Data are presented as the means ± s.e.m. and were analysed using the Mann-Whitney U-test; n = 20 (HF/HS) and n = 20 (HF/HS + PRPE). *p < 0.05. (**e**) Caloric restriction (CR) protocol: mice were fed *ad libitum* for 180 days with either the HF/HS or HF/HS + PRPE diet, and then a 30% reduction in energy intake was performed for an additional 120 days. Changes in the body weight of mice in each group are shown. Data are presented as the means ± s.e.m. and were analysed by using one-way ANOVA, n = 20 (HF/HS) and n = 20 (HF/HS + PRPE). ***p < 0.001. (**f**) eWAT cholesterol and cholesterol oxide content was assessed by GC/MS in fasted mice fed the HF/HS or HF/HS + PRPE diet for 180 days (upper panel, before CR) and after 120 days of caloric restriction (lower panel, after CR). Data are presented as the means ± s.e.m. and were analysed by using the Mann-Whitney U-test; n = 20 (HF/HS) and n = 20 (HF/HS + PRPE) before and after CR. ***p < 0.001.

Although the total fatty acid and oxidized fatty acid contents per adipose tissue weight did not differ when the HF/HS diet was supplemented with or without PRPE (Fig. 6a–c), the adipose tissue from mice fed the HF/HS diet supplemented with PRPE contained lower levels of endotoxins (Fig. 6d), cholesterol and cholesterol oxides (Fig. 6f, upper panels). Caloric restriction (CR) in obese mice from days 180 to 300 markedly reduced the body weight in a similar manner, regardless of whether the diet was supplemented with PRPE (Fig. 6e). After 120 days of caloric restriction (day 300), the levels of cholesterol and cholesterol oxides (per gram of adipose tissue) in the adipose tissue of non-supplemented mice were significantly decreased as the body weight was reduced by 30% (Fig. 6f, lower panels). In non-supplemented obese mice, the levels of cholesterol and cholesterol oxides were substantially reduced after body weight loss and no longer differed from the levels measured in PRPE-supplemented obese mice (Fig. 6f). In PRPE-supplemented mice, the low cholesterol and cholesterol oxide contents in the adipose tissue were independent of caloric restriction and weight (Fig. 6e,f). Based on these data, excess cholesterol and cholesterol oxide levels in HF/HS-fed mice are only be mobilized during body weight loss. A morphological analysis of the hypertrophic adipose tissue of obese mice fed a diet supplemented with or without PRPE revealed that PRPE did not affect adipose tissue hypertrophy and the adipocyte size (Fig. 4a–c), but markedly reduced the number of infiltrating macrophages (Fig. 4d). Accordingly, the expression of adipocyte-specific genes, *i.e*., PPARγ, aP2, perilipin1 and adiponectin, did not change (not shown), whereas the expression of macrophage markers was markedly reduced by the PRPE treatment (Fig. 4e).

### Immune-inflammatory processes are downregulated by PRPE

**Figure 7:**
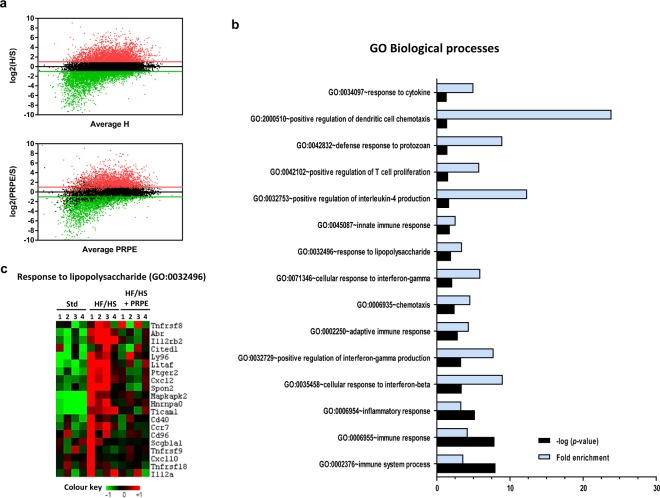
HF/HS diet supplementation with PRPE normalizes HF/HS-induced changes in the expression of genes associated with immune-inflammatory processes. (**a**) High-throughput RNA sequencing (RNA-seq) was performed on mRNA samples isolated from the eWAT of mice fed the Std (n = 4), HF/HS (n = 4) or HF/HS + PRPE (n = 4) diet for 180 days. The results are presented as fragments per kilobase of exon per million fragments mapped (FKPM (log2 values), fold changes compared to the Std (S) group) and were plotted against the average FKPM for the HF/HS (H) or HF/HS + PRPE group (PRPE). Green and red lines indicate the cutoff value for fold changes (≥2) and for differential expression (FDR < 0.05). Upper panel: Genes that were upregulated or downregulated by at least 2-fold by the HF/HS diet compared to the standard chow (Std) are labelled in red and green, respectively. Genes with similar expression are labelled in black. Lower panel: Among the 7713 genes that were significantly upregulated by the HF/HS diet (fold changes ≥2), 709 genes were significantly downregulated by PRPE supplementation. (**b**) Gene ontology analysis was performed on genes that were significantly upregulated by the HF/HS diet and significantly downregulated by PRPE supplementation. The top 15 GO biological processes are shown, along with the –log(*p*-value) and fold enrichment of significant terms. (**c**) Cluster of genes involved in the lipopolysaccharide response (GO: 0032496). Data are presented as FKPM values.

Transcriptomic analyses were performed on adipose tissues from mice fed the standard chow (Std) or HF/HS diet, which was supplemented with or without PRPE. Analyses of the RNA-seq data showed that among the 7713 genes that were significantly upregulated by the HS/HS diet compared to the Std diet (fold change > 2, FDR < 0.05), 709 genes were significantly downregulated by the PRPE supplementation (Fig. 7a). Gene Ontology analyses of biological processes revealed that the functional enrichment of genes associated with immune and inflammatory processes in mice fed the HF/HS diet was driven towards normal levels by PRPE (Fig. 7b), particularly for genes involved in the response to lipopolysaccharides (Fig. 7c).

## Discussion

**Figure 8:**
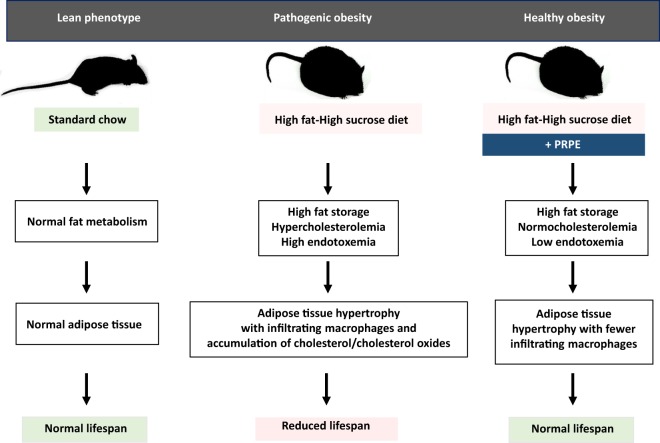
Graphical abstract. Compared to chow-fed mice with a lean phenotype, normal fat metabolism, normal adipose tissue and normal lifespan, consumption of the high-fat/high sucrose diet (HF/HS) was associated with increased fat storage, cholesterolaemia and endotoxaemia, as well as the recruitment of proinflammatory immune cells and the accumulation of cholesterol and cholesterol oxides in hypertrophic eWAT. This “unhealthy” obese phenotype was associated with a reduced lifespan. In contrast, supplementation of the HF/HS diet with the polyphenol-rich plant extract led to a substantial reduction in the number of infiltrating immune cells and a normalization of the lifespan, despite persistent adipose tissue hypertrophy. Importantly, the “healthy” obese phenotype observed after antioxidant supplementation was associated with a normalization of cholesterolaemia and endotoxaemia. The figure was entirely drawn by VA and LL.

Here, we describe a healthy obesity phenotype in an experimental animal model that is phylogenetically similar to humans. As shown in the graphical abstract (Fig. 8), mice that were chronically fed a high-fat/high-sucrose (HF/HS) diet exhibited hyperglycaemia, hypercholesterolaemia, increased oxidative stress and endotoxaemia. Compared to control mice fed normal chow, the expanded adipose tissue of HF/HS-fed mice was mainly characterized by enlarged adipocytes, the infiltration of numerous macrophages and the accumulation of higher levels of cholesterol and cholesterol oxides. As a measure of the overall lifetime health status and as the ultimate outcome of pathogenic obesity, the median lifespan was reduced by 36% in HF/HS-fed mice compared to healthy, lean mice. Here, despite the persistence of the obesity trait and adipose tissue hypertrophy, supplementation of the HF/HS diet with a polyphenol-rich plant extract (PRPE) normalized the plasma lipid and lipopolysaccharide parameters, prevented macrophage recruitment, and reduced cholesterol and cholesterol oxide accumulation in the adipose tissue of obese mice. Most importantly, the healthier metabolic phenotype of PRPE-supplemented obese mice, as compared to non-supplemented obese mice, was supported by their extended median lifespan, which was similar to control, lean mice. The maximal lifespan remained unchanged, suggesting that it mainly depended on the genetics of the mouse strain.

Although many studies have addressed the deleterious consequences of obesity and adipose tissue dysfunction, fat mass accrual may not be pathogenic in a systematic manner, prompting the concept of healthy obesity^1^. Thus, some obese individuals are considered metabolically healthy, whereas some non-obese subjects show several features of metabolic syndrome^17^. As expected from earlier studies^18^, cholesterol levels in the plasma and adipose tissue were significantly increased in C57BL/6Rj mice fed a HF/HS diet compared to lean mice fed normal chow in the present study. Blood lipid and adipose tissue abnormalities were suppressed, and the median lifespan was extended in obese mice when the HF/HS diet was supplemented with a PRPE composed of flavonols, hydroxycinnamic acids and ascorbic acid. Importantly, the beneficial effects of PRPE were independent of body weight and fat mass, which remained abnormally and equally elevated regardless of whether the diet contained PRPE. This finding provides direct support for the healthy obesity concept.

Consistent with previous studies^11,19–21^, both morphological and gene expression analyses revealed increased numbers of immune cells surrounding adipocytes, particularly macrophages, in the adipose tissue of obese mice fed the HF/HS diet. Immune cells participate in chronic inflammation^22^, and obese mice with a genetic immune or inflammation deficiency do not show metabolic dysregulation^23–25^, particularly when activity of the TLR4 endotoxin receptor is impaired^25,26^. In fact, immune cell recruitment to adipose tissues has been shown to at least partially result from endotoxaemia, which has now been recognized as a main contributor to metabolic disorders and pathogenic obesity^14,15^. In the present study, and consistent with other studies^27,28^, the plasma LPS concentration, as measured here with a direct LC-MS/MS assay^29^, was abnormally elevated in obese mice fed the HF/HS diet compared to lean mice fed normal chow. Moreover, this study is the first to report that both LPS levels and the number of immune cells in adipose tissue were significantly and concomitantly increased by the HF/HS diet. Because endotoxins have been experimentally confirmed to trigger the recruitment and accumulation of immune cells in mouse adipose tissue^30^, the diet-mediated increase in the mass LPS concentration likely directly contributed to the immune cell infiltration observed in obese mice fed the HF/HS diet in the present study. Finally, the present study is the first to show that endotoxaemia was substantially reduced when obese mice were fed the HF/HS diet supplemented with PRPE. Providing further strong support for a causal relationship between endotoxaemia and macrophage recruitment, significant decreases in the endotoxin contents in both the blood and adipose tissue of PRPE-supplemented mice translated into fewer adipose tissue-infiltrating macrophages, a weaker inflammatory profile and a longer median lifespan.

The adipose tissue of obese mice fed the HF/HS diet was a major site of cholesterol storage, which is known to depend on cholesterol biosynthesis, serum cholesterol concentrations, and adiposity^31–34^. Concomitantly with the increased macrophage number and cholesterol accumulation, adipose tissue from HF/HS-fed mice accumulated potentially harmful cholesterol derivatives in the present study. This change is likely to reflect a reactive oxygen species (ROS)-mediated increase in macrophage recruitment and cholesterol auto-oxidation through non-enzymatic processes that are known to occur mainly at position 7 of the cholesterol molecule, as shown in the present study^35–37^. Macrophage accumulation, cholesterol production and cholesterol oxidation could be reduced by PRPE in the present study in the absence of any change in adipocyte size. This finding contradicts earlier studies reporting that the adipocyte size, macrophage accumulation in adipose tissues and ROS production vary simultaneously in a coordinated manner^38^. Our results are the first to show that adipocyte expansion and health hazards may not be directly related. In other words, in addition to adipocyte hypertrophy, the accumulation of cholesterol derivatives in adipose tissue would behave similarly to the toxic, persistent organic pollutants that were previously shown to be mobilized and released as subjects lose weight^39^, thus contributing to increased mortality after weight loss in this setting^40^. In our hands, the release of toxic cholesterol derivatives from adipose tissue occurred rapidly during weight loss induced by caloric restriction or ageing. In other words, both the initial accumulation of immune cells and toxic lipids in the adipose tissue and their subsequent release later in life might constitute the two phases of a unique process contributing to health hazards during weight loss.

In previous studies, supplementation of the diet with various antioxidants (including vitamins, fish oils, red wine and red wine derivatives) has been reported to reduce metabolic diseases^41^. Although polyphenolic compounds were reported to suppress adipose tissue inflammation and to improve obesity-associated disorders^42^, their beneficial effects on reducing oxidative stress and preventing the accumulation of potentially toxic lipid derivatives in tissues were inconsistent. The present study is the first to consistently report that supplementation of the HF/HS diet with PRPE significantly reduced the circulating level of the MDA lipid peroxidation marker, substantially reduced the levels of mobilizable cholesterol and cholesterol oxides in the adipose tissue, and substantially increased median lifespan. The beneficial impact of PRPE might be directly related to the documented ability of the polyphenolic compounds to prevent the deleterious effect of the HF/HS diet on inducing obesity, which is mostly characterized by cholesterol synthesis and accumulation^43–45^, ROS production and oxidative stress^46^, and nonenzymatic cholesterol oxidation^47^. In the present study, the substantial alterations in cholesterol oxide levels in the adipose tissue of HF/HS-fed mice were prominent and leading contributors to the deleterious effects because they are well-known to behave as cytotoxic and proinflammatory molecules^48^, are involved in a number of metabolic and inflammatory diseases^49^, and classically accompany abnormal cholesterol accumulation in tissues^50^.

Because obesity may contribute to a shorter lifespan^51–54^, the reduction in the median lifespan of obese mice fed the HF/HS diet in the present study was expected. The median lifespan is considered the ultimate marker of the degradation of the overall lifelong health status, which is only estimated by measuring the levels of biological parameters, such as body weight, glycaemia, cholesterolaemia and endotoxaemia. However, roles for body fat accrual *per se* and the resulting overweight status as independent determinants of lifespan were challenged by several previous observations. Intentional weight loss is not always associated with a health benefit^54–56^, a decreased lifespan observed after the consumption of a diet high in saturated fat is not related to body weight^46^, and calorie-restricted ob/ob mice have longer lives than wild-type mice fed *ad libitum*, despite the presence of approximately twice as much body fat^57^. In this context, the present study provides new insights into the complex relationship between the adipose tissue and lifespan. Actually, the relationship appears to depend on the adipose tissue composition and inflammation rather than on fat mass and adiposity, since beneficial effects of PRPE were independent of body weight and fat mass, which remained abnormally and equally elevated whether the diet contained PRPE. Consistent with a recent study^16^, outcomes of the present study indicate that reducing harmful, immune-inflammatory fat depots in adipose tissue might be as important as weight loss *per se* and should be actually considered an emerging target for improving health and lifespan. Caloric restriction and resveratrol were previously reported as experimental nongenetic methods to increase lifespan, and the changes were mainly attributed to reduced oxidative stress, which occurred without a body weight reduction^9,58–60^.

Here, we established a novel, and effective formulation to prevent metabolic disorders and to increase the median lifespan in obese subjects, with accumulating evidence in favour of key and beneficial roles for this formulation in preventing adipose oxidation and inflammation.

## Methods

### Animals

Six-week-old C57BL/6Rj male mice were purchased from Janvier Labs (Saint-Berthevin, France). All mice were housed and maintained at 22–24 °C on a 12-h light-dark cycle in a designated pathogen-free area accredited by the Federation of Laboratory Animal Science Associations (FELASA). All experiments involving mice were performed in accordance with the institutional guidelines and were approved by the University of Burgundy’s Ethics Committee on the Use of Laboratory Animals (associated protocol n° 4711). Animals were provided *ad libitum* access to a chow diet prior to initiating the study.

### Diets

For all animal studies, mice (13 to 20 per group) were matched according to plasma insulin and cholesterol levels and body weight prior to beginning the dietary interventions. When mice were 8 to 10 weeks old, they were randomly assigned to receive one of the following diets (Research Diets): chow diet (Std, D01060501), high-fat/high-sucrose diet (HF/HS, D03062301) or high-fat/high-sucrose diet supplemented with a polyphenol-rich plant extract (HF/HS + PRPE, D10062602). A detailed description of the compositions of the diets is provided in Table 1.

For caloric restriction, eight- to 10-week-old C57BL/6Rj male mice (n = 80) were randomly assigned the HF/HS (n = 40) or the HF/HS + PRPE diet (n = 40). After 180 days of feeding on the diet *ad libitum*, mice were further subdivided into 2 subgroups (n = 20 mice per group) in which the diet was supplemented with or without the tested PRPE at 180 days of feeding. For the remaining two groups, daily energy food intake was reduced by 30% over a 120-day period.

### Polyphenol-rich plant extract

Active XXS inside^®^ supplied by Lara-Spiral (21560 Couternon, France) includes antioxidant polyphenol-rich extracts from food plants (Asteraceae*, Lactuca*; Liliaceae*, Allium cepa;* Lamiaceae*, Ajuga;* and Verbenaceae*, Lippia*). XXS™ complies with regulations on food contaminants and banned and prohibited substances. The polyphenol-rich plant extract (PRPE) was analysed by using HPLC with an HPLC-200 (Perkin Elmer, Massachusetts, USA) coupled to a diode array detector. Separation was performed using a linear gradient on a Licrospher 100 RP-18 column (150 × 4.6 mm; 5 µm) (Merck, Massachusetts, USA) maintained at 27 °C. The quaternary pump was connected to mobile phases (A), consisting of pH 2.2 water containing trifluoroacetic acid, and (B), consisting of acetonitrile. The following gradient was used in the present study: 0–5 min, 0% B and 5–50 min, 0–55% B. Twenty microliters of samples were injected into the chromatograph and the flow rate was 1 mL/min. Simultaneous monitoring was performed at 280 and 345 nm. Based on the results, the PRPE contains 25 g of polyphenols/100 g of extract (w/w), including 10 g/100 g of flavonoids (quercetin, 3.4 g/100 g; and glycosylated quercetin, 5.1 g/100 g) and 15 g/100 g of hydroxycinnamic acids (chlorogenic acid, 0.5 g/100 g; chicoric acid, 5.1 g/100 g; and phenylpropanoid caffeic acid glycosides, 5.0 g/100 g).

### Mouse sample handling

After a 4-h fast (each diet group at each experimental time point), mice were anaesthetized by isoflurane inhalation. A cardiac puncture was performed using a 25 G needle, and a minimum of 500 µL of blood were collected in a heparinized tube. Plasma was obtained by centrifuging blood samples (8000 rpm for 10 min). Plasma samples were aliquoted and stored at −80 °C until further analysis. The epididymal white adipose tissue (eWAT) was harvested from fasted mice, weighed, snap-frozen in liquid nitrogen and stored at −80 °C until further analysis. For immunohistochemical analyses, eWAT sections were prepared from freshly harvested tissues that had been fixed with 4% paraformaldehyde (VWR, Fontenay-sous-Bois, France) for 24 h before being embedded in paraffin.

### RNA extraction and real-time quantitative PCR analysis

Epididymal white adipose tissue (eWAT) was harvested from fasted mice, weighed, immediately snap frozen (immersion in liquid nitrogen) and stored at −80 °C until further analysis. Total RNA was extracted from eWAT samples by using the RNeasy Lipid Tissue Mini kit (Qiagen, Courtaboeuf, France) according to the manufacturer’s instructions and included a DNase treatment step. RNA concentrations were quantified using the NanoDrop 1000 spectrophotometer (Thermo Scientific, Illkirch, France), and 500 ng of RNA were then reverse transcribed into cDNAs using M-MLV reverse transcriptase, random primers and RNaseOUT inhibitor (Invitrogen, Thermo Scientific, Illkirch, France). The expression of each cDNA was quantified by using real-time PCR with the Power SYBR Green PCR Master Mix (Applied Biosystems, Warrington, UK) on a Viia7 Real-Time PCR system (Applied Biosystems,Warrington, UK). Relative mRNA levels were determined by using the ΔΔCt method and normalized to the expression of the mouse 36B4 gene. The following primer sequences were used: m-*Emr1* forward 5′-CTTTGGCTATGGGCTTCCAGTC-3′, reverse 5′-GCAAGGAGGACAGAGTTTATCGTG-3′; m-*CD68* forward 5′-CCAATTCAGGGTGGAAGAAA-3′, reverse 5′-CTCGGGCTCTGATGTAGGTC-3′; m-*CD11b* forward 5′-GTTTGTTGAAGGCATTTCCC-3′, reverse 5′-ATTCGGTGATCCCTTGGATT-3′; m-*Arg1* forward 5′-TGGCTTGCGAGACGTAGAC-3′, reverse 5′-GCTCAGGTGAATCGGCCTTTT-3′; m-*Mcp1* forward 5′-CCCAATGAGTAGGCTGGAGA-3′, reverse 5′-TCTGGACCCATTCCTTCTTG-3′ and m-*36B4* forward 5′-ATGGGTACAAGCGCGTCCTG-3′, reverse 5′- GCCTTGACCTTTTCAGTAAG -3′.

### Lipidomic analyses by using mass spectrometry

Epididymal white adipose tissue and plasma were harvested from fasted mice, immediately snap frozen (immersion in liquid nitrogen) and stored at −80 °C until further analysis. Total lipids were extracted from eWAT (50–150 mg) by using the Bligh and Dyer method^61^. Briefly, tissues were first extracted with saline (500 µL) and chloroform/methanol 2/1 v/v (3750 µL) in 10-mL glass tubes for 1 h at room temperature on a rotary agitator (20 rpm). Chloroform (1.25 mL) was then added and the extraction proceeded for 1 h. Finally, water (1.75 ml) was added, providing two phases. After centrifugation (1500 g for 5 min), the lower organic phase was collected and stored at −20 °C.

Hydroxyoctadecadienoic acids (HODEs), hydroxyeicosatetraenoic acids (HETEs) and 7-hydroxycholesterols (7α and 7β) were quantified using the method described by Yoshida *et al*.^62^, with some modifications. Briefly, dried lipids from eWAT (equivalent to 25 mg of eWAT) or plasma samples (200 µL) were solubilized with MeOH (500 µL) containing butylated hydroxytoluene (50 mg/mL) and saline (200 µL). Each sample was spiked with an internal standard mixture containing 7α-hydroxycholesterol-d7 (200 ng), 7β-hydroxycholesterol-d7 (200 ng) (Avanti Polar Lipids, Alabaster, Alabama, USA), 13-HODE-d4 (80 ng), 9-HODE-d4 (40 ng) and 15-HETE-d8 (40 ng) (Cayman, Ann Arbor, Michigan, USA). Samples were then mixed with a NaBH_4_/NaCl (5.25 g of NaCl at 9 g/L + 39.375 mg of NaBH_4_) solution and incubated for 5 min at room temperature before saponification for 40 min at 40 °C with a MeOH/KOH (1 M) solution. Lipids were then extracted with 2 mL of acetic acid (10%, Sigma Aldrich) and 5 mL of CHCl_3_/ethyl acetate (4/1, v/v) (Acros Organics, Fisher Scientific, Illkirch, France). After centrifugation, the organic phase was divided in two equal aliquots for the analyses of sterols and HODEs/HETEs, respectively.

Oxysterols were analysed by using GC-MS as described below. After solvent evaporation under a vacuum, sterols were further solubilized with 100 µL of a mixture of N,O-bis(trimethylsilyl)trifluoroacetamide (BSTFA) and trimethylcholorosilane (TMCS) (4/1, v/v) and incubated at 80 °C for 1 h. After evaporation, the residue was dissolved in 100 µL of hexane and one microliter was injected onto an HP-5MS 30 m x 250 µm column. The GC-MS analysis was conducted on a 6890 GC chromatograph equipped with an HP7683 injector and a 5973 C mass-selective detector (Agilent Technologies, Les Ulis, France) operating with an electronic impact mode source setup at 70 eV. The ions used for the analysis were: 7α- and 7β-hydroxycholesterol, 456.2 m/z and 7α- and 7β-hydroxycholesterol (d7), 463.2 m/z. Calibration curves were obtained using authentic standards (10–640 ng) extracted with the same method used for samples.

HODEs and HETEs were quantified using a 1290-LC 6490-QqQ system (Agilent Technologies, Les Ulis, France). Briefly, after evaporating the organic phase under a vacuum, lipids were solubilized with 100 µL of a 70/30 v/v methanol/water mixture and 4 µL were injected onto a Zorbax Eclipse Plus C18 column (2.1 × 100 mm, 1.8 µm column, Agilent Technologies, Les Ulis, France). Separation was achieved at a flow rate of 0.3 mL/min at 30 °C using the following linear gradient of 5 mM ammonium acetate (solvent A) and acetonitrile/methanol (95/5, v/v) (solvent B): 23% B for 6.5 min, up to 50% B in 8.5 min, up to 52% B in 3 min and maintained at 52% for 5 min. Data were acquired in negative multiple reaction monitoring (MRM) mode (source temperature: 200 °C, nebulizer gas flow rate: 15 L/min, sheath gas flow rate: 11 L/min, temperature: 250 °C, capillary: 3500 V, collision energy: 18 V and 10 V for HODEs and HETEs respectively). Transitions of 299.4 → 198.0, 295.0 → 195.2, 295.0 → 183.1, 295.2 → 170.9, 319 → 219.2, 319 → 167.2, 319 → 163.2, 319 → 155.2, 319 → 115.2 and 327.4 → 226.1 were used to quantify 13-HODE-d4, 13-HODE, (12 + 10)-HODE, 9-HODE, 15-HETE, 11-HETE, 12-HETE, 8-HETE, 5-HETE and 15-HETE-d8, respectively. Calibration curves were obtained using authentic standards (5–320 ng) extracted with the same method used for samples.

Total fatty acids analysis. Samples (20 µL of plasma or total lipids from an equivalent of 0.25 mg of eWAT) were analysed as previously described^63^.

Total cholesterol analysis. Plasma (20 µL) or total lipids levels in an equivalent of 1.0 mg of eWAT were spiked with 10 µg and 1 µg of the internal standard epi-coprostanol, respectively, and analysed as previously described^63^.

LPS concentrations analysis. The eWAT (30–50 mg) and plasma (50 µL) samples were first hydrolysed for 3 h at 90 °C with 300 µL of 8 M HCl to determine LPS concentrations. Thereafter, lipids were extracted with 600 µL of water and 5 mL of a hexane/ethylacetate mixture (2:3, v/v) (Thermo Fisher, Illkirch, France). After centrifugation, the hexanic phase was collected, further dried under a vacuum and then the residue was dissolved with 200 µL of EtOH (Thermo Fisher, Illkirch, France) and transferred to injection vials. After evaporation, samples were dissolved in 50 µL of EtOH before analysis by using LC-MS/MS. 3-OH C14:0 and 3-OH C13:0 (Matreya, Clinisciences, Nanterre, France) were used as external and internal standards, respectively. Fatty acids were separated using a QQQ 6490 triple quadruple mass spectrometer (Agilent Technologies, Les Ulis, France) equipped with a 50-mm Zorbax SBC18 column (Agilent Technologies, Les Ulis, France) set to 45 °C and with a JetStream ESI source in the negative mode (gas temperature: 290 °C, gas flow rate: 19 L/min, nebulizer: 20 psi, sheath gas temperature: 175 °C, sheath gas flow rate: 12 L/min, and capillary voltage: 2,000 V). Two microliters of each sample were injected. A 13-min elution gradient was established as follows: from 0 to 0.5 min, 55% of A (water, ammonium formate 5 mM, formic acid 0.1%) and 45% of B (acetonitrile/water (95/5, v/v), ammonium formate 5 mM, formic acid 0.1%); from 3 to 8 min, 100% of B; and from 8.10 to 13 min, 55% of A and 45% of B. The flow rate was maintained at 0.4 mL/min throughout the gradient elution process. The mass spectrometer was set in MRM mode. A calibration curve was constructed with increasing concentrations of the external standard 3-OH C14:0. 3-OH C14:0 levels in samples were quantified by calculating the 3-OH C14:0/3-OH C13:0 ratio by using the calibration curve.

Chromatograms were analysed with the Agilent MassHunter Workstation software, and calibration curves were created using authentic standards extracted with the same method used for samples.

### Blood biochemical analyses

Fasting blood samples were collected from the mice in each group after 0, 1.5, 3 and 6 months of diet consumption via cardiac puncture into heparin-containing tubes. Plasma was separated by centrifugation and stored at −80 °C until further analysis. Plasma total glucose, cholesterol and triglyceride levels were quantified with enzymatic methods using a Konelab Prime 30i diagnostic system (Thermo Scientific, Illkirch, France) and the reagents and instructions provided by the manufacturer. The size distributions of plasma lipoproteins, particularly the HDL subfraction, were analysed as previously described^64^. Briefly, the size distributions of plasma lipoprotein subfractions were determined using non-denaturing polyacrylamide gradient gel electrophoresis with SpiraGel™ (Spiral Laboratories, Couternon, France). A 1.5–25% gradient SpiraGel™ (82 × 82 × 2.6 mm) enables the simultaneous determination of the size distributions of both plasma LDL and HDL fractions. This high-resolution gel is capable of resolving HDL subfractions 3c (7.21–7.76), 3b (7.76–8.17 nm), 3a (8.17–8.77 nm), 2a (8.77–9.71 nm), 2b (9.71–12.9 nm), and an HDL subfraction with mean diameter greater than 12.9 nm. Solutions with a density of 1.21 were prepared by adding KBr, and lipoproteins were isolated by using ultracentrifugation (5.5 h at 100000 rpm). Plasma subfractionation was performed at 150 V for 10 h at 4–10 °C, followed by 180 V for 11 h (3 500 V.h), in Tris buffer (90 mM Tris HCl, 80 mM boric acid, and 3 mM EDTA, pH 8.3). After electrophoresis, gels were stained with a 0.04% Coomassie brilliant blue G-250 solution and destained. Densitometry was used to measure the peak diameter of HDL subfractions in scanned gels. Peak HDL particle diameters were determined from calibration curves of 1000/molecular diameter of standards (nm) using 2^nd^ order polynomial equations. Standards, including a High Molecular Weight kit (GE Healthcare Life Sciences, Little Chalfont, UK) and calibrated LDL (25.5 and 27.0 nm), were run on every gel.

Lipid contents of fast protein liquid chromatography (FPLC)-separated lipoprotein fractions were also quantified by using ESI-MS/MS at Synelvia SAS (Prologue Biotech, Labege, France).

### Determination of overall antioxidant defences

The overall antioxidant defence potential was monitored in fresh EDTA-treated blood using the KRL™ biological test (Spiral Laboratories, Couternon, France). The principle of the KRL test is to subject whole blood or erythrocyte suspensions to a temperature-controlled free radical reaction. The KRL analysis was conducted according to the manufacturer’s recommendations. Briefly, blood samples were mixed with an isotonic saline solution to measure the levels of organic free radicals produced at 37°C following the thermal decomposition of a solution of 2,2′–azobis (2-amidinopropane) dihydrochloride (AAPH). Similar to physiological conditions, both extracellular and intracellular antioxidant defences contribute to maintaining the cell integrity until haemolysis. Erythrocyte resistance was recorded using a 96-well microplate reader by measuring the decrease in absorbance at 620 nm. The resistance of whole blood and red blood cells to free radical attack is presented as the time required to achieve 50% haemolysis (half-haemolysis time, T_1/2_ in minutes)^65^ (US patent n°s 5,135,850 A and 20060234329 A1).

### Plasma malondialdehyde assay

The total MDA level in plasma was determined by using HPLC on an HPLC-200 (Perkin Elmer, Massachusetts, USA) with fluorimetric detection (515/553 nm) after the derivatization of MDA with 2-thiobarbituric acid (TBA) using the method described by Moselhy *et al*.^66^. Briefly, plasma samples (250 µL) were shaken for 30 min in a boiling water bath in the presence of 800 µL of 0.44 M phosphoric acid, 100 µL of 0.05% butylated hydroxytoluene (BHT) in EtOH and 200 µL of 0.8% thiobarbituric acid. The TBA-MDA adduct was separated on a 4.6-mm reverse-phase Licrospher RP-8 250 column (Merck, Massachusetts, USA) and quantified by measuring the fluorescence (ex = 515 nm; em = 553 nm). The mobile phase was a mixture of acetonitrile and 0.05 M phosphate buffer, pH 6.8 (40:60) under isocratic conditions at 45 °C; the flow rate was 1.25 mL/min, and 20 µL of each sample were injected onto the column. MDA concentrations were determined from calibration curves prepared using 1,1,3,3-tetramethoxypropane.

### EchoMRI

The body composition, which is presented as the percentage of fat mass and lean mass, was determined using EchoMRI (Echo Medical Systems, Houston, Texas, USA).

### F4/80 immunostaining

Paraffin-embedded eWAT tissues were serially sectioned into 5 µm slices. Immunohistochemistry (IHC) was performed after deparaffinization, rehydration, and antigen retrieval by heating in 10 mM sodium citrate buffer (pH 6, 95 °C). Sections were then saturated in a 3% BSA solution containing 3% hydrogen peroxide to block endogenous peroxidase activity. After saturation, sections were incubated with a rat anti-mouse F4/80 antibody (1/100; AbD Serotec, MCA497) for 1 h at room temperature. Sections were further incubated with ImmPress anti-rat Ig (Vector Laboratories, MP7444). IHC staining in sections was visualized using a NovaRed revelation kit (Vector Lab, SK4800) and a Leica DM 6000 microscope (Leica Microsystems). Negative controls were performed by incubating sections without the primary antibody. All sections were counterstained with haematoxylin (Leica, 380156E). Positive F4/80 infiltrates were counted in 5 different fields per section and two different sections per group. Adipocyte size distribution frequencies and mean sizes were evaluated in stained sections.

### mRNA sequencing analysis

For high-throughput mRNA sequencing (RNA-seq), total RNA was extracted from eWAT samples with the RNeasy Lipid Tissue Mini kit (Qiagen, Courtaboeuf, France) according to the manufacturer’s instructions and included a DNAse treatment step. RNA-seq was performed by the Platform of Transfer in Cancer Biology of the Georges-François Leclerc Center (Dijon, France). Briefly, mRNA was selected from 1 µg of total RNA with the NEBNext Poly(A) mRNA magnetic isolation module (NEB). The RNA-seq library was prepared with 100 ng of RNA depleted of rRNA with a NEBNext Ultra RNA library kit for Illumina sequencing according to the manufacturer’s instructions (New England BioLabs, Evry, France), and sequencing was performed on a NextSeq500 device (Illumina, San Diego, USA). The libraries were sequenced with pair-end 76-base pair “reads”. RNA-seq data were analysed for differentially expressed genes and transcripts using the TopHat and Cufflinks informatics procedures^67^. An unsupervised hierarchical clustering analysis was performed using Gene Cluster 3.0 software and viewed with the Treeview viewer. Values were normalized and centred on the mean. Hierarchical clustering was performed with Euclidean distance measure and complete linkage analyses. Upregulated genes are shown in red and downregulated genes are shown in green. Genes that were significantly upregulated by at least 2-fold (FDR < 0.05) in mice fed the HF/HS diet compared to mice fed the standard chow and significantly downregulated by the PRPE formulation were further used for gene ontology and enrichment analyses using the Database for Annotation, Visualization, and Integrated Discovery (DAVID v.6.8) software. The threshold was set according to the modified Fisher Exact *p*-value (EASE score) ≤ 0.05.

### Statistical analyses

All animals were included in the analyses. Statistical analyses were performed with Prism software 6 (GraphPad software). Data are presented as the means ± standard errors of the means (s.e.m). Continuous data were compared using the Mann-Whitney U-test, multiple Student’s *t*-tests, the Kruskal-Wallis test with Dunn’s post hoc analysis or one-way ANOVA followed by Tukey’s multiple comparison test, as appropriate, after confirming a normal distribution and homogeneity of variance. All *p*-values are two-tailed; *p*-values less than 0.05 were considered significant (*p < 0.05, **p < 0.01 and ***p < 0.001). For survival curves, data were analysed with StatView 5.0 software using the Peto-Peto Prentice test.

## Data Availability

The authors declare that all data supporting the findings of this study are available within the article or from the corresponding authors upon reasonable request.

## Code Availability

The RNA-seq data have been deposited in the NCBI Gene Expression Omnibus (GEO) (http://ncbi.nlm.nih.gov/geo) with accession number GSE120748.

Visit https://www.ncbi.nlm.nih.gov/geo/query/acc.cgi?acc = GSE120748 and enter token gbidqeiaxjurvat into the box to review GEO accession number GSE120748.
